# Innovations in Community: Leveraging Community Networks to Reduce Social Isolation and Loneliness

**DOI:** 10.1093/geroni/igaf122.1573

**Published:** 2025-12-31

**Authors:** Amy Eisenstein, Jillian Racoosin Kornmeier

## Abstract

Meaningful connection is a fundamental part of what constitutes a good life at any age. Just as the causes of social isolation and loneliness are multi-faceted, and in many cases interrelated, addressing these issues is equally complex. Existing services and supports within the community can lay the groundwork to build innovative solutions. This symposium will bring together three community-centered research projects examining implementation of innovative approaches to address social connection in community settings. Presenters will share lessons learned from working within the community to address this issue. The first presenter will describe the initial steps of a community-engaged initiative to adapt and implement a Social Prescribing Decision Support Tool for use across diverse settings, and will discuss lessons learned from community partnerships necessary to make this work successful. The second presenter will discuss the role of Parks and Recreation professionals and the creation of a Healthy Aging in Parks Framework (HAIPF), along with plans and preliminary work to test the implementation of the HAIPF in four communities across the country. The third presenter will discuss implementation outcomes of Connect ME: a Brief Behavioral Activation intervention to increase social connectedness among homebound Meals on Wheels clients living in rural Maine. The session will be pulled together with a discussion on how we can take these lessons learned to continue to implement and disseminate innovative programs to reduce loneliness and create meaningful connections within existing community networks.

